# Recombinant PAL/PilE/FlaA DNA vaccine provides protective immunity against *Legionella pneumophila* in BALB/c mice

**DOI:** 10.1186/s12896-020-00620-3

**Published:** 2020-05-18

**Authors:** Yingying Chen, Zehui Yang, Ying Dong, Yu Chen

**Affiliations:** Department of Pulmonary and Critical Care Medicine, Shengjing Hospital of China Medical University, 36 Sanhao Street, Shenyang, 110004 People’s Republic of China

**Keywords:** *Legionella pneumophila*, DNA vaccine, PAL, PilE, FlaA

## Abstract

**Background:**

*Legionella pneumophila* (*L.pneumophila*), a Gram-negative small microorganism, causes hospital-acquired pneumonia especially in immunocompromised patients. Vaccination may be an effective method for preventing *L.pneumophila* infection. Therefore, it is necessary to develop a better vaccine against this disease. In this study, we developed a recombinant peptidoglycan-associated lipoprotein (PAL)/type IV pilin (PilE)/lagellin (FlaA) DNA vaccine and evaluated its immunogenicity and efficacy to protect against *L.pneumophila* infection.

**Results:**

According to the results, the expression of PAL, PilE, FlaA proteins and PAL/PilE/FlaA fusion protein in 293 cells was confirmed. Immunization with PAL/PilE/FlaA DNA vaccine resulted in highest IgG titer and strongest cytotoxic T-lymphocyte (CTL) response. Furthermore, the histopathological changes in lung tissues of mice challenged with a lethal dose of *L.pneumophila* were alleviated by PAL/PilE/FlaA DNA vaccine immunization. The production of T-helper-1 (Th1) cytokines (IFNγ, TGF-α, and IL-12), and Th2 cytokines (IL-4 and IL-10) were promoted in PAL/PilE/FlaA DNA vaccine group. Finally, immunization with PAL/PilE/FlaA vaccine raised the survival rate of mice to 100% after challenging with a lethal dose of *L.pneumophila* for 10 consecutive days.

**Conclusions:**

Our study suggests that the newly developed PAL/PilE/FlaA DNA vaccine stimulates strong humoral and cellular immune responses and may be a potential intervention on *L.pneumophila* infection.

## Background

*Legionella pneumophila* (*L.pneumophila*) is a Gram-negative small microorganism, which is widely found in nature and man-made water systems [1, 2]. The sporadic, epidemic, or hospital infection of *L.pneumophila* can be fatal, especially in immunocompromised patients [3, 4]. If the patients do not receive timely and correct diagnosis and treatment, the mortality rate of *L.pneumophila* infection can be as high as 50% [5]. Currently, there are limited effective measures to prevent *L.pneumophila* infection. Therefore, developing an effective, safe vaccine with no toxic side effects to fight against *L.pneumophila* infection is of significance.

Early studies have found that animals artificially infected with *L.pneumophila* could generate a humoral or cell mediated immune response [6, 7]. In addition, compared with the mono-antigen vaccine, a higher humoral immunity and stronger protective immunity are induced by the recombinant multi-antigen vaccine to protect against *L.pneumophila* infection [8, 9]. *L.pneumophila* contains multiple virulence factors, such as peptidoglycan-associated lipoprotein (PAL), lagellin (FlaA), and type IV pilin (PilE). PAL is a 19 kDa outer membrane lipoprotein, and as a species distinctive immunodominant component can be served as a diagnostic indicator for *L.pneumophila* infection [10]. FlaA protein is a key component of *L.pneumophila* flagella. The flagella can enhance the invasion ability of bacteria, which promotes the infection to host cells [11]. Moreover, study suggested that FlaA could play crucial roles in the protective immunity against lethal dose infection of *L.pneumophila* in mice via stimulating T-cell-mediated immune reaction [12]. PilE protein has been demonstrated to facilitate the adhesion between bacteria and their host cells, and is closely related to the DNA transformation of *L.pneumophila* [13]. So far, the effect of recombinant PAL/PilE/FlaA vaccine against *L.pneumophila* infection has not been determined.

Thus, in the present study we selected PAL, PilE, and FlaA for the candidates to construct a new recombinant DNA vaccine and investigated its immunogenicity and protective efficacy against *L.pneumophila* infection in mice.

## Results

### Construction of recombinant plasmids and expression of recombinant proteins in 293 cells

**Fig. 1:**
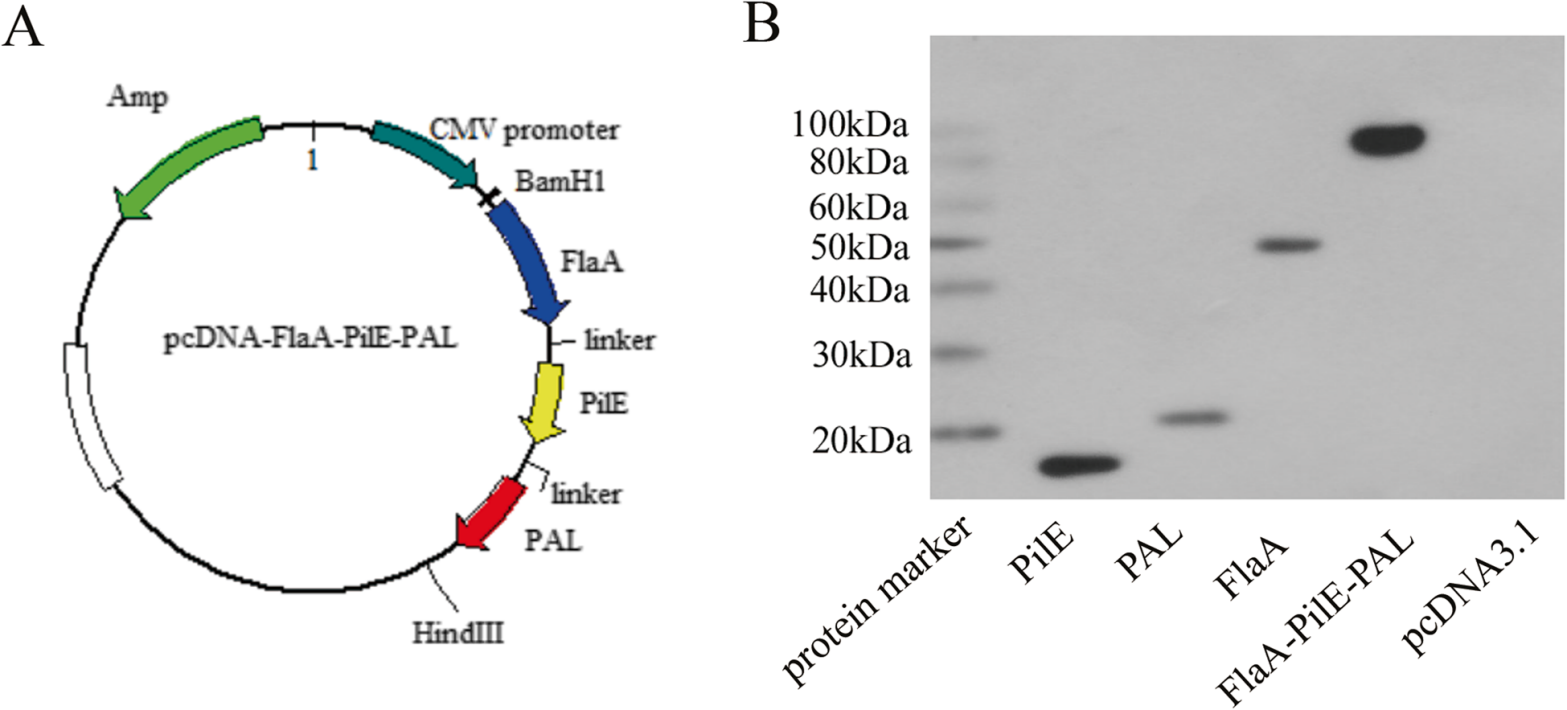
Construction of recombinant expression vector and verification of recombinant protein expression in 293 cells. **a** Construction maps of recombinant pcDNA-FlaA-PilE-PAL. **b** 293 cells were transiently transfected with pcDNA-FlaA, pcDNA-PilE, pcDNA -PAL, pcDNA-FlaA-PilE-PAL or pcDNA 3.1 for 72 h, then the cell lysates were subjected to Western blotting assay using rabbit *Legionella pneumophila* polyclonal antibody

The full-length gene sequences of PAL, PilE, and FlaA were synthesized and separately cloned into the pcDNA3.1 vector to generate recombinant plasmids of pcPAL, pcPilE, pcFlaA, and pcPAL/PilE/FlaA for expressing PAL, PilE, FlaA, or the fusion protein PAL/PilE/FlaA, respectively. To verify the expression of these proteins in eukaryotic cells, these recombinant plasmids were transfected into 293 cells. As shown in Fig. 1b, the Western blotting result confirmed the expression of PilE (about 15 kDa), PAL (about 19 kDa), FlaA (about 34 kDa), and PAL/PilE/FlaA (about 70 kDa).

### DNA vaccines induced humoral immune response in mice

**Fig. 2:**
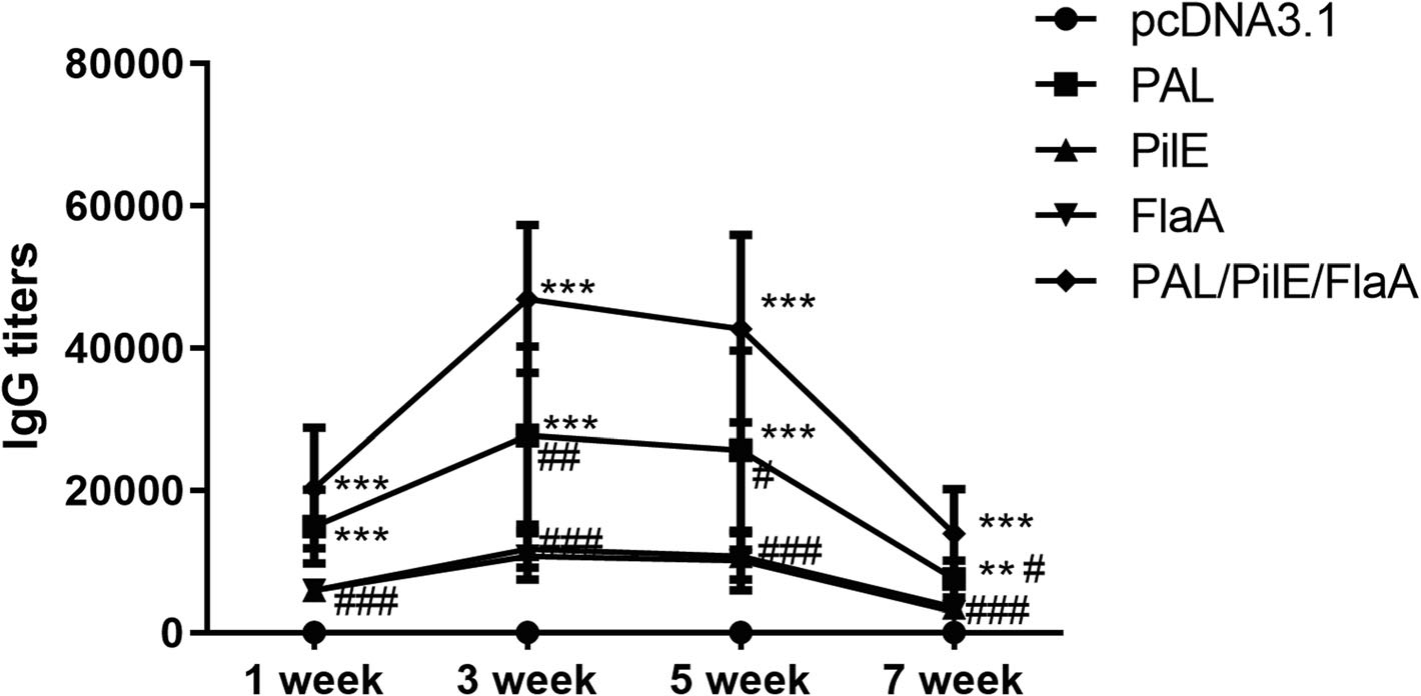
Humoral immune responses of the DNA vaccines in the immunized mice. 50 μg of DNA vaccines were biweekly intramuscularly injected into the mice for three times. The IgG titers of multiples groups were detected by ELISA at 1, 3, 5, and 7 weeks after the last immunization. All data were expressed as mean ± SD (*n* = 6). ^**^, *p* < 0.01. ^***^, *p* < 0.001 versus the pcDNA3.1 group. ^#^, *p* < 0.05, ^##^, *p* < 0.01, ^###^, *p* < 0.001 versus the PAL/PiLE/FLaA group

To assess the recombinant DNA vaccines-induced humoral immune response in mice, the IgG titers were detected by ELISA. As presented in Fig. 2, the IgG titers were gradually increased from 1 week to 5 weeks after the enhanced immunization, which were greatly declined at 7 weeks after the enhanced immunization. Among all these recombinant DNA vaccines, the IgG titer in PAL/PilE/FlaA group was significantly increased, and these groups from high to low, in turn, is PAL/PilE/FlaA, PAL, PilE and FlaA (Numerical values are shown in Table 1). In the pcDNA3.1 group, the IgG titer was undetectable. Thus, these results suggested that DNA vaccine pcPAL/PilE/FlaA induced a stronger humoral immune response in mice.

**Table 1:** IgG titers at 1, 3, 5, and 7 weeks after the last immunization

Group	1 week	3 weeks	5 weeks	7 weeks
pcDNA3.1	0 ± 0	0 ± 0	0 ± 0	0 ± 0
PAL	14,933 ± 5226^***^	27,733 ± 12585^***/##^	25,600 ± 14022^***/#^	7467 ± 2613^**/#^
PiLE	5867 ± 1306^###^	11,733 ± 2613^###^	10,667 ± 3305^###^	3467 ± 1573^###^
FLaA	5867 ± 1306^###^	10,667 ± 3305^###^	10,133 ± 4253^###^	2933 ± 653^###^
PAL/PiLE/FLaA	20,267 ± 8507^***^	46,933 ± 10451^***^	42,667 ± 13220^***^	13,867 ± 6292^***^

^**^, *p* < 0.01. ^***^, *p* < 0.001 versus the pcDNA3.1 group. ^#^, *p* < 0.05, ^##^, *p* < 0.01, ^###^, *p* < 0.001 versus the PAL/PiLE/FLaA group

### DNA vaccines induced CTL response in mice

**Fig. 3:**
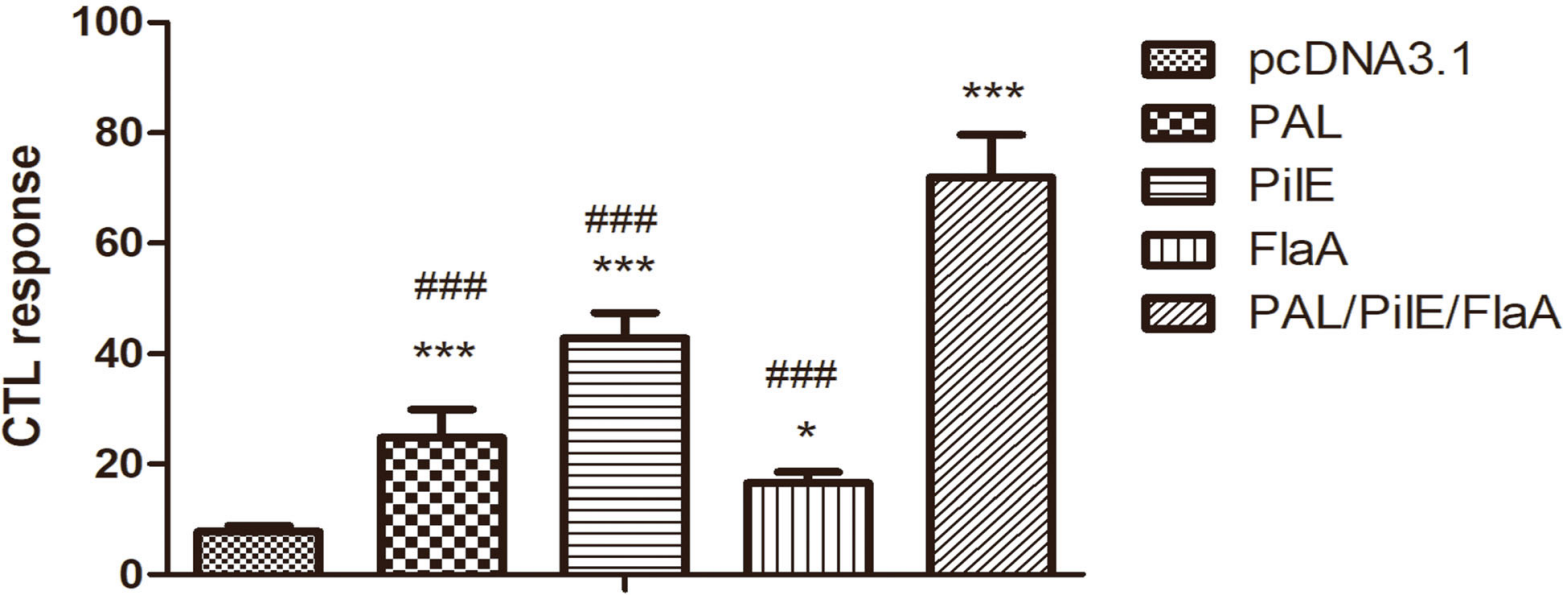
The cytotoxic T-lymphocyte (CTL) response in mice immunized with the DNA vaccines. At 7 weeks after the last immunization, the spleen lymphocytes were separated. The CTL response was detected by MTT assay. All data were expressed as mean ± SD (*n* = 6). ^*^, *p* < 0.05. ^***^, *p* < 0.001 versus the pcDNA3.1 group. ^###^, *p* < 0.001 versus the PAL/PiLE/FLaA group

The spleen lymphocytes were isolated from mice to determine CTL response. As assessed by MTT assay and shown in Fig. 3, the CTL response was stronger in PAL, PilE, FlaA, and PAL/PilE/FlaA groups compared with pcDNA3.1 group. Among these recombinant DNA vaccine groups, PAL/PilE/FlaA group showed strongest CTL response (Numerical values are shown in Table 2).

**Table 2:** The cytotoxic T-lymphocyte (CTL) response in mice immunized with the DNA vaccines

Group	pcDNA3.1	PAL	PiLE	FLaA	PAL/PiLE/FLaA
Cytotoxicity	7.784 ± 1.096	24.793 ± 5.085^***/###^	42.887 ± 4.537^***/###^	16.541 ± 2.040^*/###^	71.932 ± 7.752^***^

^*^, *p* < 0.05. ^***^, *p* < 0.001 versus the pcDNA3.1 group. ^###^, *p* < 0.001 versus the PAL/PiLE/FLaA group

### Immunization with recombinant PAL/PilE/FlaA DNA vaccine protected mice against L.pneumophila challenge

**Fig. 4:**
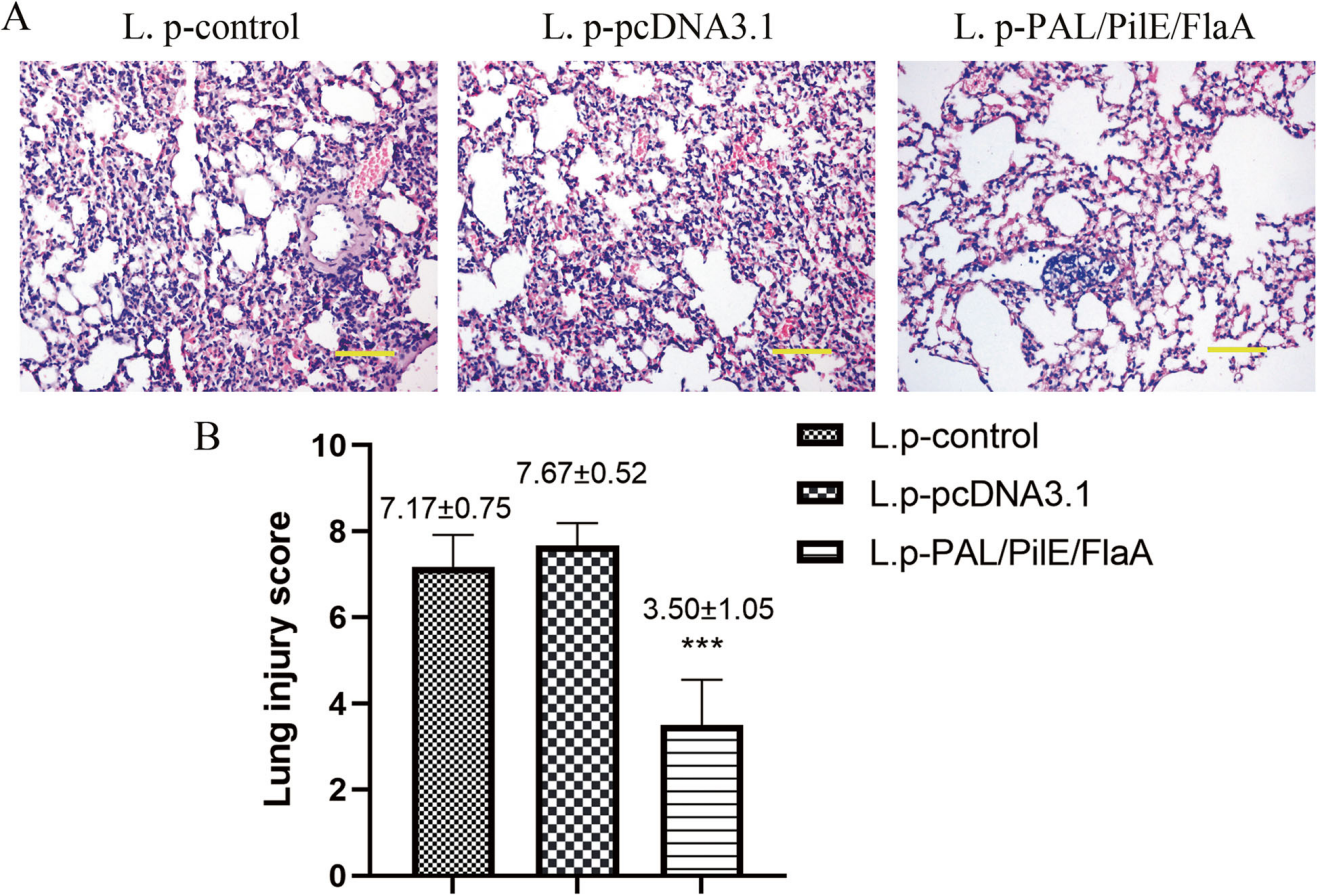
The histological morphologic changes of lungs in mice challenged with a lethal dose of *Legionella pneumophila*. **a** The lung sections from different groups were stained with hematoxylin and eosin (HE). Scale bar = 100 μm. **b** Lung injury score was shown. All data were expressed as mean ± SD (*n* = 6). ^***^P < 0.001 versus the L.p-pcDNA3.1 group

**Fig. 5:**
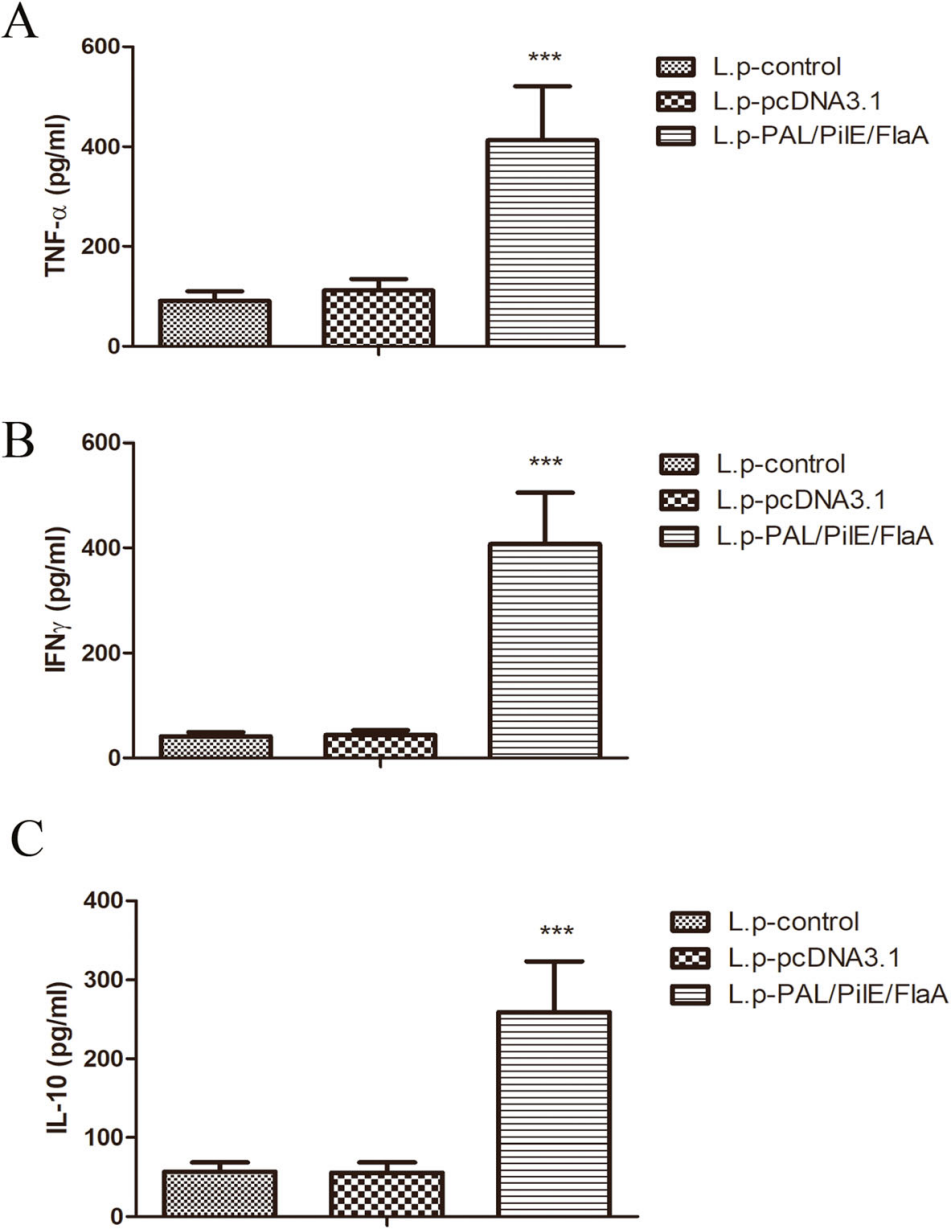
The cytokine levels of serum samples collected from mice at 16 h after a lethal challenge with *Legionella pneumophila*. The TNF-α (**a**), IFNγ (**b**), and IL-10 (**c**) levels were evaluated by ELISA kits. All data were expressed as mean ± SD (*n* = 6). ^***^P < 0.001 versus the L.p-pcDNA3.1 group

**Fig. 6:**
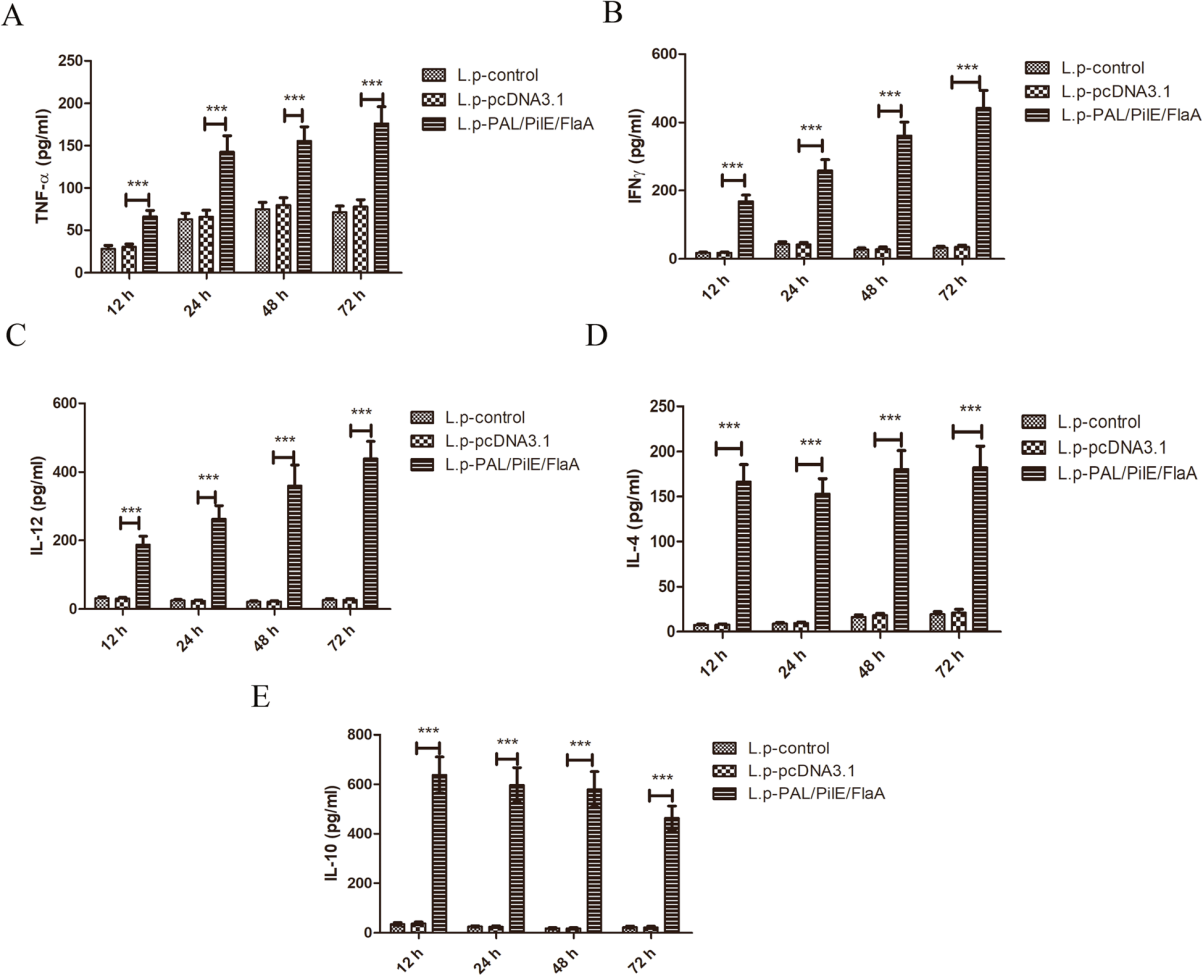
The production levels of cytokines from splenocytes extracted from mice at 16 h after a lethal challenge with *Legionella pneumophila*. The supernatants of splenocytes were collected after culture for 12, 24, 48, and 72 h. The levels of TNF-α (**a**), IFNγ (**b**), IL-12 (**c**), IL-4 (**d**), and IL-10 (**e**) were determined by ELISA kits. All data were expressed as mean ± SD (*n* = 3). ***P < 0.001 versus the L.p-pcDNA3.1 group

**Fig. 7:**
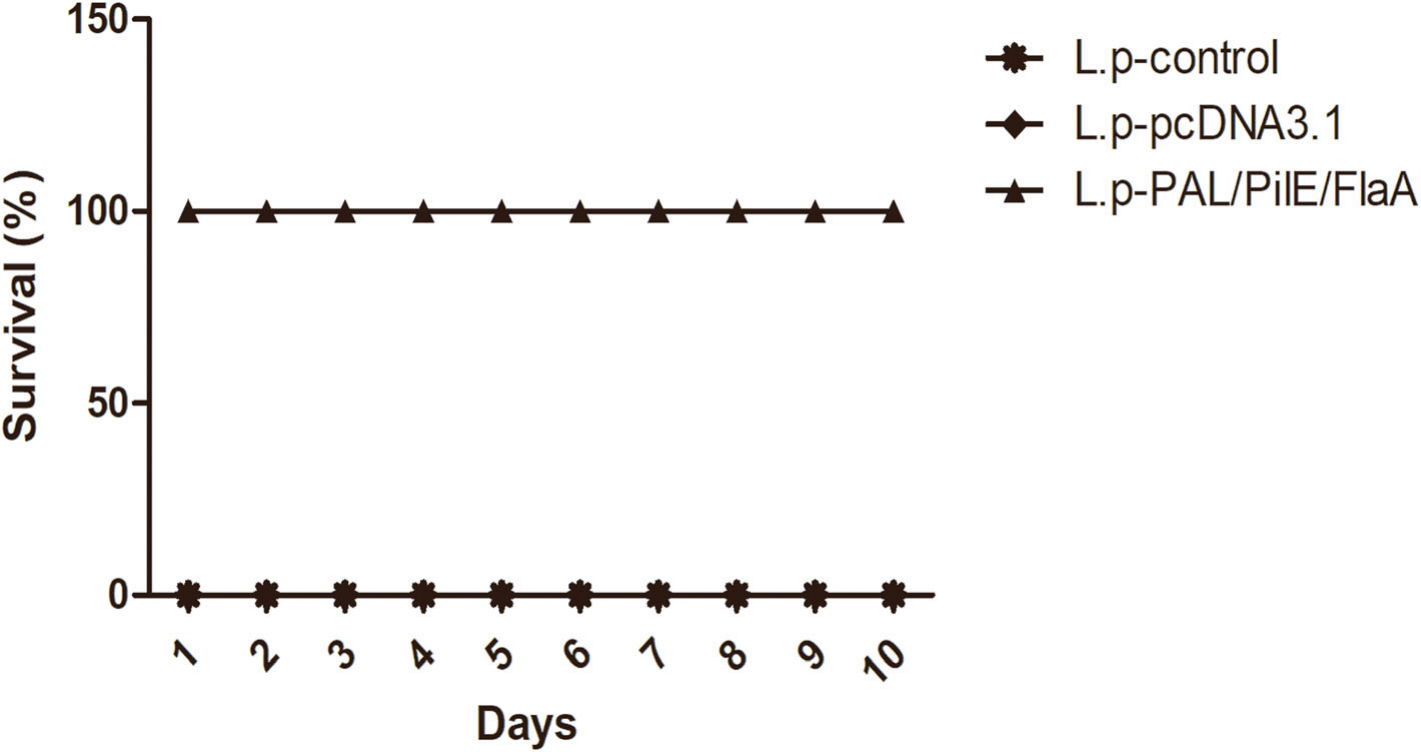
Protective immunity in mice after a lethal dose challenge with *Legionella pneumophila*. The survival rate of mice for 10 days after a lethal dose challenge with *Legionella pneumophila* was shown (*n* = 10)

To further investigate PAL/PilE/FlaA DNA vaccine-induced protective immunity in mice, the histopathological changes in lung tissues of mice after challenging with a lethal dose of *L.pneumophila* were observed by HE staining. As illustrated in Fig. 4a&b, there were significant inflammatory cell infiltration and destruction of alveolar tissues in the lung tissues of L.pneumophila-infected mice. However, in the lung tissues of mice immunized with PAL/PilE/FlaA DNA vaccine, the inflammatory cell infiltration was obviously restrained. Moreover, the cytokine response was determined by ELISA. As presented in Fig. 5a-c, the serum levels of TNF-α, IFNγ, and IL-10 were significantly up-regulated in PAL/PilE/FlaA group, as compared with control or pcDNA3.1 group (Numerical values are shown in Table 3). In the supernatant of splenocyte cultures of mice challenged with a lethal dose of *L.pneumophila*, the levels of TNF-α, IFNγ, IL-12, IL-4 and IL-10 were remarkably enhanced in PAL/PilE/FlaA group after culture for 12, 24, 48, and 72 h (Fig. 6a-e, numerical values in Table 4). The survival rate of mice after infection with *L.pneumophila* was monitored for 10 consecutive days. As shown in Fig. 7, the survival rate of mice immunized with PAL/PilE/FlaA DNA vaccine was 100% up to 10 days after infection with *L.pneumophila;* whereas, there were no living mice in control and pcDNA3.1 non-immunized groups from 1 day to 10 days. All the above results indicated that PAL/PilE/FlaA DNA vaccine could effectively prevent *L.pneumophila* infection in mice.

**Table 3:** The cytokine levels of serum samples collected from mice at 16 h after a lethal challenge with *Legionella pneumophila*

	L.p-control	L.p-pcDNA3.1	L.p-PAL/PiLE/FLaA
TNF-α	91.219 ± 19.774	112.061 ± 23.189	413.019 ± 107.533^***^
IFNγ	41.047 ± 8.446	43.582 ± 9.246	407.293 ± 98.131^***^
IL-10	57.249 ± 11.516	55.869 ± 12.854	259.314 ± 63.963^***^

^***^, *p* < 0.001 versus the L.p-pcDNA3.1 group

**Table 4:** The cytokine levels of splenocytes extracted from mice after a lethal challenge with *Legionella pneumophila*

		L.p-control	L.p-pcDNA3.1	L.p-PAL/PiLE/FLaA
TNF-α	12 h	28.464 ± 3.656	30.544 ± 3.160	66.426 ± 6.966^***^
	24 h	63.061 ± 6.975	66.022 ± 7.707	142.812 ± 18.872^***^
	48 h	74.966 ± 8.033	79.856 ± 8.523	155.792 ± 16.642^***^
	72 h	71.350 ± 7.257	78.119 ± 8.014	176.366 ± 19.816^***^
IFNγ	12 h	17.882 ± 2.151	18.063 ± 1.958	168.855 ± 17.771^***^
	24 h	42.829 ± 6.708	41.573 ± 6.185	259.489 ± 31.379^***^
	48 h	27.588 ± 4.677	28.726 ± 5.612	361.732 ± 39.412^***^
	72 h	32.878 ± 3.567	35.138 ± 4.788	442.975 ± 51.127^***^
IL-10	12 h	35.905 ± 5.921	38.285 ± 6.257	638.272 ± 72.853^***^
	24 h	26.358 ± 2.762	25.189 ± 3.560	597.381 ± 70.460^***^
	48 h	18.722 ± 2.776	18.359 ± 3.338	580.414 ± 71.515^***^
	72 h	23.453 ± 4.065	22.517 ± 4.267	464.455 ± 47.797^***^
	12 h	7.545 ± 0.848	7.866 ± 0.832	166.512 ± 19.016^***^
	24 h	9.129 ± 1.099	9.616 ± 1.083	153.189 ± 16.667^***^
IL-4	48 h	16.399 ± 2.269	18.371 ± 2.041	180.543 ± 20.530^***^
	72 h	19.566 ± 2.546	21.151 ± 3.770	182.311 ± 23.732^***^
	12 h	32.346 ± 3.307	31.066 ± 3.123	187.781 ± 24.391^***^
	24 h	25.581 ± 2.879	24.311 ± 2.506	263.204 ± 38.746^***^
IL-12	48 h	21.841 ± 2.484	22.015 ± 2.566	360.088 ± 60.515^***^
	72 h	27.231 ± 3.618	27.220 ± 3.528	439.624 ± 50.110^***^

^***^, *p* < 0.001 versus the L.p-pcDNA3.1 group

## Discussion

In this study, we developed a recombinant PAL/PilE/FlaA DNA vaccine to protect against *L.pneumophila* infection in mice. The results suggested that immunization with recombinant PAL/PilE/FlaA DNA vaccine successfully induced humoral and cellular immunity, alleviated lung inflammation and enhanced the survival rate of *L.pneumophila*-challenged mice.

Recombinant DNA vaccine provides the possibility for production of antigen protein with high purity, which may replace inactivated vaccine and attenuated live vaccine because of its high security and easy production features [14, 15]. Human gene therapy is a clinical reality. As reported, the NIH and the FDA have submitted proposals to eliminate RAC review and reporting requirements to the NIH for human gene-therapy protocols [16]. In addition, the recombinant protein produced by *Escherichia coli* could not exactly reflect the native structure of bacterial protein, so it is not ideal to evaluate protective efficacy of recombinant protein vaccine in rabbits [17]. After immunization with DNA vaccine, the endogenous antigen protein with natural conformation can be produced by cells within the body, which induces humoral and cellular immunity just like pathogen infection [18]. Compared with recombinant protein vaccine, the titer of produced high affinity antibody is 100–1000 times higher after the injection of DNA vaccine [19]. Therefore, compared with traditional vaccines, DNA vaccine has the characteristics of strong and long immune responses, and no virulence reversion. A previous study has indicated that pcDip/pilE DNA vaccine is effective to protect against *L.pneumophila* infection [8]. In our study, we constructed a new DNA vaccine with three protective antigens and evaluated its immune effects.

Since gene synthesis is an effective method to obtain DNA template [20], it is used to construct DNA vaccine, which avoids the pathogen culture and lowers the risk for pathogen infection. Chen et al. synthesized the optimized coding sequence of CHA5 to build CHA5 DNA vaccine that could induce broad protection against H5N1 influenza viruses [21]. In a recent study, the cDNA sequences encoding full-length Ebola GP and VP40 were synthesized to construct the DNA vaccine, which induced specific humoral and cellular immune responses in mice [22]. In this study, the cDNA sequences of PAL, PilE, and FlaA were synthesized and cloned into pcDNA3.1 vector. The expression of PAL, PilE, FlaA and PAL/PilE/FlaA fusion proteins were confirmed in 293 cells after transfecting with recombinant plasmids, which provided a good foundation for the ongoing study.

The ideal vaccine should be an efficient inducer of both humoral and cellular immune responses. To observe the humoral immune responses induced by these recombinant DNA vaccines, we performed ELISA to detect the titer of specific IgG antibody after three times of immunization. According to our results, immunization with PAL, PilE, FlaA and PAL/PilE/FlaA recombinant DNA vaccines could significantly enhance the IgG titer. The results also suggested that PAL/PilE/FlaA recombinant DNA vaccine exhibited the most obvious effect. The strong CTL response demonstrated that the PAL, PilE, and FlaA proteins played pivotal roles in antigen presentation and subsequent induction of cellular immune response. Our results indicated that the CTL response was strongest in PAL/PilE/FlaA recombinant DNA vaccine group compared with that in PAL, PilE, or FlaA group. All these results proved that PAL/PilE/FlaA recombinant DNA vaccine could induce higher humoral and cellular immune responses, so we next evaluated the protective immunity of PAL/PilE/FlaA DNA vaccine against a lethal challenge with *L.pneumophila.*

Helper T cell cytokines are a kind of mediators that have extensively biological activities. T-helper-1 (Th1) cell derived cytokines such as IFNγ, TGF-α, and IL-12 can promote the synthesis of IgG2a and enhance CTL response, which play crucial roles in cellular immune response. While Th2 cell cytokines such as IL-4 and IL-10 contribute to B cell proliferation and IgG1 synthesis, which mainly induce humoral immune response [23]. The balance between Th1 and Th2 cytokines maintains immune homeostasis. The type of immune responses and efficacy of vaccines can be evaluated through the detection of secreted cytokines after vaccination [24, 25]. In the present study, we detected the levels of Th1 cytokines IFNγ, TGF-α, and IL-12, and Th2 cytokines IL-4 and IL-10 at 16 h after *L.pneumophila infection.* We found that the serum levels of TNF-α, IFNγ, IL-10 were increased, and in the supernatant of splenocytes the levels of TNF-α, IFNγ, IL-12, IL-4 and IL-10 were remarkably enhanced after immunization with PAL/PilE/FlaA DNA vaccine. Thus, PAL/PilE/FlaA DNA vaccine induced both Th1 and Th2 immune responses in mice. Moreover, the survival and histopathological changes in lung tissues of mice were improved by the immunization with PAL/PilE/FlaA vaccine. Therefore, protective immunity was induced by PAL/PilE/FlaA DNA vaccine against *L.pneumophila* infection in mice.

The inoculation methods may affect the safety of DNA vaccine. It has been demonstrated that intramuscular injection, gene gun bombardment, and electroporation can be safe inoculation methods for DNA vaccine [26–31]. Protocols of DNA immunization by electroporation, besides improving per se both arms of the immune response [30, 31], are widely in use in pre-clinical studies and have been approved and are on-going in phase I and II clinical trials. The efficiency of naked DNA delivery can be improved dramatically when combined with in vivo electroporation [32] and is being used clinically in advanced trials treating cervical dysplasia (NCT01304524, NCT03180684, NCT03185013). The popularity of naked DNA has been relatively stable, and it is the most popular nonviral system used in clinical trials [33]. Based on these studies, the risk of PAL/PilE/FlaA vector integration into the host genome was considered very low. Because no poisonous side effects were observed in mice after injection of our DNA vaccine, the possibility of PAL/PilE/FlaA vector integration into the host genome was not assessed in this work.

## Conclusion

Recombinant PAL/PilE/FlaA DNA vaccine shows higher potential to enhance the IgG titer and induce strong CTL response, compared with each comprising protein, indicating stronger humoral and cellular immune responses are stimulated. Moreover, the recombinant DNA vaccine can effectively protects against a lethal challenge with the *L.pneumophila* in mice. The DNA vaccine PAL/PilE/FlaA may be useful in vaccination against *L. pneumophila* infection.

## Methods

### Animals

Six-to-eight-week-old female BALB/c mice (weight about 20 g) were purchased from Liaoning changsheng biotechnology co. Ltd. The mice were housed under a specific pathogen free condition at 22 ± 1 °C, humidity of 45–55%, and a 12 h light/dark cycle, with free access to food and water.

### Bacterial strains, media and growth conditions

Bacteria of *L. pneumophila* serogroup 1 (American Type Culture Collection, USA; no. 35133) were cultured on buffered charcoal-yeast extract agar with buffered charcoal yeast extract (BCYE) (Merck, Germany) in a candle urn at 37 °C with humidified atmosphere and collected with phosphate buffered saline (PBS). After washing in sterile PBS and centrifugation at 4 °C, the bacteria were diluted to a proper concentration.

### DNA vaccine construction, purification, and expression in mammalian cells

The cDNA sequences encoding full-length PAL (Gene ID: 19833609), PilE (Gene ID: 19833480), and FlaA (Gene ID: 19832905) antigens were synthesized by Sangon Biotech Co., Ltd. (Shanghai, China) and cloned into the corresponding sites of the eukaryotic expression vector pcDNA3.1 (Invitrogen, Carlsbad, USA). The first linker between FlaA and PilE is (G_4_S)_3_. The second linker between PilE and PAL is EASPPGE. The obtained recombinant plasmids, named as pcPAL, pcPilE, pcFlaA, and pcPAL/PilE/FlaA, respectively, were identified by DNA sequencing. The plasmid profile for pcPAL/PilE/FlaA is shown in Fig. 1a. The recombinant plasmids were transformed into competent *E. coli* BL21. After culture in LB medium at 37 °C overnight, the *E. coli* BL21 was induced by 1 mM isopropyl-D-thiogalactopyranoside (IPTG) for 4 h to express His-tagged fusion proteins. Fusion proteins were purified by HisTrap affinity columns (GE Healthcare) and dialysis, which were used as antigen for subsequent antibody detection. 293 cells were purchased from Zhong Qiao Xin Zhou Biotechnology Co., Ltd. (RZQ0002, Shanghai, China) and were maintained in minimum essential medium (MEM, Gibco, USA) containing 10% fetal bovine serum (Hyclone, USA) at 37 °C under 5.0% CO_2_ atmosphere. To analyze the fusion proteins, 293 cells were transfected with the recombinant plasmids pcPAL, pcPilE, pcFlaA, and pcPAL/PilE/FlaA, respectively, using lipofectamine 2000 (Invitrogen, USA) according to the manufacturer’s instructions. After the transfection for 72 h, the protein expression was detected by Western blotting as described below.

### Western blotting

293 cells were lysed in RIPA (Solarbio, China) containing 1 mM PMSF (Solarbio). The protein concentration was evaluated by BCA Protein Assay Kit (Solarbio). Subsequently, 20 μg protein sample was subjected to sodium dodecyl sulfate polyacrylamide gel electrophoresis and transferred to polyvinylidene difluoride membranes (Millipore, USA). After blocking in 5% skimmed milk, the membranes were incubated with Rabbit anti-*L.pneumophila* polyclonal antibody (1:500, MyBioSource, USA) at 4 °C overnight. Then the membranes were incubated with Goat Anti-rabbit IgG/HRP antibody (1:3000, Solarbio) at 37 °C for 1 h. The bands were visualized by ECL solution (Solarbio).

### Immunization of mice

The BALB/c mice randomly divided into five groups (*n* = 6 per group, total 30) were separately immunized by pcDNA3.1, pcPAL, pcPilE, pcFlaA, and pcPAL/PilE/FlaA. Briefly, the mice were intramuscularly injected with 50 μg pcDNA3.1, 50 μg pcPAL, 50 μg pcPilE, 50 μg pcFlaA, and 50 μg pcPAL/PilE/FlaA, respectively. Two weeks and 4 weeks after the immunization, the mice were re-injected with the above DNA vaccines at the same dose to enhance immunization. At 1, 3, 5, and 7 weeks after the enhanced immunization, the serum samples were collected and stored at − 70 °C. The mice were euthanized by receiving an overdose of pentobarbital sodium (200 mg/kg, i.p.), and the spleen lymphocytes were isolated from mice at 7 weeks after the enhanced immunization for further experiments.

### Antibody detection

The total immunoglobulin G (IgG) titers were determined by indirect enzyme-linked immunosorbent assay (ELISA). Briefly, the 96-well plates were coated with 100 μl recombinant PAL/PilE/FlaA antigen (0.1 μg per well) at 4 °C overnight. After washing in PBST buffer for three times, the plates were blocked in 5% skimmed milk at 37 °C for 2 h. Then 100 μl serial dilutions of serum samples were added to each well and incubated at 37 °C for 1 h. Then the plates were washed in PBST buffer for three times and incubated with HRP-labeled Goat Anti-Mouse IgG (1:250, Beyotime, China) at 37 °C for 1 h. The plates were then incubated with 200 μl TMB Chromogen Solution (Beyotime) at 37 °C for 20 min in the dark. To terminate the reaction, 50 μl of 2 M H2SO4 was added to each well. The results were detected at 450 nm by a microplate reader (BioTek, USA).

### Measurement of the cytotoxic T-lymphocyte (CTL) response

CTL response was detected by the methyl-thiazolyl-tetrazolium (MTT) method as previously described [8]. Briefly, the isolated spleen lymphocytes (5 × 10^6^ /ml, effector cells) from the immunized mice were mixed with the cells expressing PAL, PilE, FlaA, and PAL/PilE/FlaA (5 × 10^5^ /ml, target cells), respectively, and then seeded into 96-well plates. The single cultured spleen lymphocytes or PAL, PilE, FlaA, and PAL/PilE/FlaA positive expressing cells were used as the effector control or target control. After culture for 56 h, the cells in each group were incubated with 0.5 mg/ml MTT at 37 °C for 4 h. After discarding the supernatant, each well was added with 150 μl DMSO. The absorbance at 570 nm was detected by a microplate reader. The CTL response was evaluated as the following formula: CTL = [1-(A_570 effector_ -A_570 effector control_)]/A_570 target control_× 100%.

### Studies of protective immunity

The BALB/c mice were randomly divided into three groups (*n* = 16 per group, total 48): control group, pcDNA3.1 group, and pc PAL/PilE/FlaA group, and intramuscularly injected with equal volume of PBS, 50 μg pcDNA3.1, or 50 μg pcPAL/PilE/FlaA, respectively. The mice were re-injected with the above DNA vaccines at the same dose to enhance immunization at 2 weeks and 4 weeks after the immunization. Two weeks after the enhanced immunization, the mice were intravenously injected with a lethal dose of *L.pneumophila* (2 × 10^7^ CFU in PBS). At 16 h after the injection of *L.pneumophila,* serum samples were collected from 6 mice in each group. Then the mice were euthanized by an overdose of pentobarbital sodium (200 mg/kg, i.p.), and the lung tissues were removed and fixed in 4% paraformaldehyde. The spleen tissues were collected for isolation of splenocytes. The remaining 10 mice in each group were monitored for another 10 days for survival analysis and euthanized at day 11.

### Hematoxylin-eosin (HE) staining

To observe the pathological changes in the lung tissues, HE staining was performed. The lung tissues were embedded in paraffin and cut into 5-μm sections. Then the sections were subjected to routine HE staining. The results were observed under a light microscope (Olympus, Japan) and the images were taken at a magnification of 200×. The alveolar edema, hemorrhage, and inflammatory infiltration were scored on a scale of 1–3 (0: absent, 1: mild, 2: moderate, 3: severe) with a maximum score of 9 [34].

### Cytokine response analysis

Cytokine levels in serum samples or the supernatants of splenocytes cultured for 12, 24, 48, and 72 h were detected by commercial ELISA kits for TNF-α, IL-12, IFNγ, IL-4, and IL-10 (USCN Business Co., Ltd., Wuhan, China), according to the manufacturer’s instructions.

### Statistical analysis

All results are shown as mean ± standard deviation (SD). One-way ANOVA followed by Bonferroni’s Multiple Comparison Test was performed to analyze data among different groups using GraphPad Prism 5 software. A *P* value of less than 0.05 was considered to be statistically significant.

## Data Availability

The datasets used and/or analysed during the current study are available from the corresponding author on reasonable request.

## Abbreviations

*L.pneumophila*: *Legionella pneumophila*
PAL: Peptidoglycan-associated lipoprotein
PilE: Type IV pilin
FlaA: Lagellin
CTL: Cytotoxic T-lymphocyte
Th1: T-helper-1
BCYE: Buffered charcoal yeast extract
PBS: Phosphate buffered saline
MEM: Minimum essential medium
IgG: Immunoglobulin G
ELISA: Enzyme-linked immunosorbent assay
MTT: Methyl-thiazolyl-tetrazolium
HE: Hematoxylin-eosin
SD: Standard deviation
